# Measuring impact of storyline engagement on health knowledge, attitudes, and norms: A digital evaluation of an online health-focused serial drama in West Africa

**DOI:** 10.7189/jogh.12.04039

**Published:** 2022-05-14

**Authors:** Philip M Massey, Matthew D Kearney, Alexandre Rideau, Adam Peterson, Jessica D Gipson, Roch A Nianogo, Marta Bornstein, Michael L Prelip, Deborah C Glik

**Affiliations:** 1 Department of Health, Human Performance, and Recreation, College of Education and Health Professions, University of Arkansas, Fayetteville, Arkansas, USA; 2 Department of Family Medicine and Community Health, Perelman School of Medicine, University of Pennsylvania, Philadelphia, Pennsylvania, USA; 3 African Health and Education Network (RAES), Dakar, Senegal; 4 Department of Biostatistics, School of Public Health, University of Michigan, Ann Arbor, Michigan, USA; 5 Department of Community Health Sciences, UCLA Fielding School of Public Health, Los Angeles, California, USA; 6 Department of Epidemiology, UCLA Fielding School of Public Health, Los Angeles, California, USA; 7 College of Public Health, The Ohio State University, Columbus, Ohio, USA

## Abstract

**Background:**

“Cest la Vie!” (CLV) is a serial drama that entertains, educates, and promotes positive health behaviors and social change for West African audiences. The purpose of this study was to evaluate if watching the CLV Season 2 series online had an impact on people’s health knowledge, attitudes, and norms, focusing on populations in francophone West Africa.

**Methods:**

Between July 2019 and October 2019, viewers of CLV and non-viewers were recruited from Facebook and YouTube. We conducted an online longitudinal cohort study that assessed changes in health knowledge, attitudes, and norms (KAN) between these groups. Participants completed a baseline survey prior to the online airing and up to three follow-up surveys corresponding to specific health stories in the series, including sexual violence, emergency contraception, and female circumcision. We used descriptive statistics to describe viewers and non-viewers, and an item response theory (IRT) analysis to identify the effect of viewing CLV on overall KAN.

**Results:**

A total of 1674 respondents participated in the study. One in four participants (23%, n = 388) had seen one of the three storylines from CLV Season 2 (ie, CLV viewers). At follow-up, viewers were more likely than non-viewers to know when to correctly use emergency contraception (*P* < 0.001) and to believe that the practice of female circumcision should end (*P* = 0.001). Compared to people who did not see CLV, viewers of the series had 26% greater odds of answering pro-health responses at follow-up about sexual assault, emergency contraception, and female circumcision. Further, the level of engagement with specific storylines was associated with a differential impact on overall outcome questions.

**Conclusions:**

As internet access continues to grow across the globe and health education materials are created and adapted for new media environments, our study provides a novel approach to examining the impact of online entertainment-education content on health knowledge, attitudes, and norms.

Broadcast radio and television dramas have used memorable storylines to engage the public and have been instrumental in changing cultural perceptions, social norms, social behaviours, and public policies that impact health [1-5]. More recently this approach has been translated to online and social media platforms [6,7]; however, few studies have focused on the impact of this relatively new yet pervasive media environment [8].

Entertainment-education (EE) continues to evolve and engage audiences around the globe [9,10]. In low- and middle-income countries (LMICs) traditional EE strategies have been leveraged for decades for health promotion and education. These strategies have shifted population perceptions, social norms, and behaviours regarding the prevention and treatment of HIV, STIs, and other infectious diseases, reproductive health, child survival, and gender violence, often with varying degrees of success [11,12]. Studies examining the impact of EE on health have found an increase in positive attitudes and beliefs, behavioural changes, and high levels of peer communication among viewers [13,14]. Pathways that facilitate changes in knowledge and behaviours, such as mediating and moderating effects, have also been explored, including audience engagement and identification with characters [15,16].

The growing availability of and desire to use digital technologies in LMICs provides an opportunity for traditional media and EE strategies to integrate online components [17]. Online content has the potential to reach audiences across regions, providing access to health education and information that can otherwise be difficult to obtain, particularly among younger populations [18]. Access to online health information can be especially valuable to young adults in sub-Saharan Africa, as it allows them to learn about often taboo topics, such as sexual and reproductive health, without having to experience potential social stigma and shame in discussing these topics with parents, health care professionals, or other adults [19]. The use of storytelling in EE to promote pro-health attitudes and behaviours, combined with the growing reach of online technologies, shows great promise in this next chapter of digital social and behavioural change communication [20].

“Cest la Vie!” (CLV) is a serial drama created in French for West African audiences. First developed in 2017 for broadcast media (ie, television and radio), CLV content entertains, educates, and promotes positive health behaviours and social change [21]. Storylines tackle complex and often taboo health topics, including sexual assault, emergency contraception, and female circumcision, as well as the daily realities that West African individuals, families, and communities experience. The drama focuses on health care professionals and patients within a health care setting, similar to the two of the longest-running medical shows in the US, *ER* and *Grey’s Anatomy* [22,23]. In addition to its television broadcast throughout West Africa, all episodes of the CLV series were made available on YouTube and Facebook in 2017, expanding its digital presence and allowing for greater audience reach.

The purpose of this study was to evaluate the impact of watching CLV Season 2 online on viewers’ knowledge, attitudes, and norms (KAN) related to various health topics discussed in the series, focusing on populations in francophone West Africa. Specifically, we address the following research questions (RQ):

RQ1: Is exposure to C’est la Vie! (CLV) online content associated with changes in overall health KAN?
RQ2: Are different storylines associated with varying levels of change in overall KAN?
RQ3: Are different storylines associated with varying levels of change in specific KAN?
RQ4: Is self-reported engagement with the storylines associated with respondents discussing the CLV series with others?

## METHODS

### Study design, setting, and participants

To evaluate the impact of watching C’est La Vie! (CLV) Season 2 online among francophone West African adults, we conducted an online longitudinal cohort study that assessed changes in health KAN associated with watching the series. Between July 2019 and October 2019, CLV viewers/media interactors and non-viewers/media interactors were recruited online using advertisements (ads) on Facebook and YouTube. Using the Facebook ad manager, we targeted recruitment ads to people 18 years or older in four countries (Senegal, Niger, Burkina Faso, or Côte d'Ivoire) and stratified recruitment into two groups – those who had already interacted with CLV content on Facebook (ie, viewers/media interactors), and those who had never interacted with CLV content on Facebook (ie, non-viewer/media interactors). To reach non-viewers, we used the “lookalike” audience feature in the Facebook ad manager, targeting people 18 years or older who were similar in their online behaviours to viewers (eg, what types of Facebook pages they were fans of or the types of content they had interacted with) except they had not interacted with CLV on Facebook. On YouTube, we posted recruitment materials to the CLV channel to recruit viewers, while also posting recruitment materials to channels that played similar content, ie, other popular web series in West Africa, to recruit non-viewers. Both paid and organic (ie, without cost) content were used to recruit individuals; paid content, such as Facebook advertisements, allowed for additional targeting criteria as previously described. Eligible participants were persons 18 years or older who lived in Senegal, Niger, Burkina Faso, or Côte d'Ivoire, all francophone West African countries. To complete enrolment, participants reviewed the study information sheet that described the study, stated participation was voluntary and that they could stop participation at any time for any reason without repercussions, that their responses would be de-identified and reported in aggregate, and that they would be compensated 2000 West African CFA franc per completed survey (US$4). After reviewing and agreeing to the study information, participants were enrolled. The study protocol was approved by an accredited Institutional Review Board committee at Drexel University.

### Data collection and management

Data collection began in July 2019 and ended in July 2020. Each participant responded to a baseline questionnaire and up to three follow-up questionnaires. The baseline questionnaire was shared with participants prior to the online release of CLV Season 2, beginning in mid-July 2019. The 36-episode CLV Season 2 series released about two episodes each week on the CLV Facebook and YouTube pages; the full season, if watched synchronously, spanned approximately six months. Follow-up questionnaires were sent to participants at the conclusion of three select storylines and were related to: 1) sexual assault (episodes 10-14, two months after baseline in October 2019); 2) emergency contraception (episodes 20-22, three months after baseline in November 2019); and 3) female circumcision (episodes 27-28, four months after baseline in December 2019). Data collection and timing were similar across the viewer and non-viewer groups, such that all participants received the follow-up surveys the same number of months after baseline, regardless of what group they were in. To accommodate study participants who may not have watched online content synchronously, as well as to maximize the number of follow-ups completed, we reached out to participants in both groups up to six months after all the episodes in Season 2 had been released, concluding data collection in July 2020.

All responses were recorded in Qualtrics software and exported to REDCap for data management [24]. Among participants who consented and provided contact information, we distributed follow-up questionnaires via email and WhatsApp. Completed follow-up responses were matched with participants’ baseline data using unique Qualtrics hyperlinks and REDCap identifiers. We reached out to participants up to three times per follow-up phase and asked them to complete follow-ups sequentially (ie, individually after each storyline). However, if a participant did not respond to one of the follow-up surveys, we continued reaching out to them and allowed them to subsequently complete prior storyline questions. Accordingly, participants could have completed pre-post data for all storyline and health outcomes questions, regardless of whether they responded sequentially, at each individual follow-up separately, or simultaneously, to multiple follow-ups. Through such an approach, we maximized our ability to evaluate pre- vs post-storyline differences.

Questionnaire items were originally developed in English and translated to French by francophone team members in Senegal, followed by back-translation to ensure the meaning and integrity of questions were consistent across language and cultural translations. Prior to the start of the study in July 2019, we piloted the questionnaire with an online sample (n = 40) recruited using the same Facebook ads and targeting metrics (ie, age, country) used in the study.

### Measures

Participant demographic attributes measured were age, sex, country, education level, religion, and urban/rural residence (see **Table 1**).

**Table 1:** Participant characteristics for viewers and non-viewers of C’est La Vie (CLV) storylines, baseline data collection July 2019 (n = 1674)*

Subject Characteristic	Overall (n = 1674)	Not seen CLV storyline (n = 1286)	Seen any CLV storyline (n = 388)
**Age**	24 (21-29)	24 (21-29)	23 (21-27)
**Sex**			
Male	673 (40%)	539 (42%)	134 (35%)
Female	1001 (60%)	747 (58%)	254 (65%)
**Country**			
Senegal	842 (50%)	610 (47%)	232 (60%)
Burkina Faso	251 (15%)	200 (16%)	51 (13%)
Niger	151 (9.0%)	131 (10%)	20 (5.2%)
Cote de d'lvoire	298 (18%)	215 (17%)	83 (21%)
Other	132 (7.9%)	130 (10%)	2 (0.5%)
**Education:**			
None	7 (0.4%)	6 (0.5%)	1 (0.3%)
Primary	21 (1.3%)	20 (1.6%)	1 (0.3%)
Middle School	45 (2.7%)	40 (3.1%)	5 (1.3%)
High School	344 (21%)	284 (22%)	60 (15%)
College	1244 (73%)	908 (71%)	316 (81%)
Other	33 (2.0%)	28 (2.2%)	5 (1.3%)
**Religion:**			
Traditionalist	20 (1.2%)	18 (1.4%)	2 (0.5%)
Catholic	318 (19%)	237 (18%)	81 (21%)
Protestant	150 (9.0%)	118 (9.2%)	32 (8.2%)
Muslim	1131 (68%)	868 (67%)	263 (68%)
None	27 (1.6%)	22 (1.7%)	5 (1.3%)
Other	28 (1.7%)	23 (1.8%)	5 (1.3%)
**Urbanicity:**			
Urban	1466 (88%)	1105 (86%)	361 (93%)
Rural	52 (3.1%)	47 (3.7%)	5 (1.3%)
Urban/Rural	156 (9.3%)	134 (10%)	22 (5.7%)

CLV – C’est La Vie

*Numbers presented as_ n (%); median (IQR).

*Key outcome*: KAN items related to storyline health topics were key outcomes of the study. Storyline health topics included sexual assault, emergency contraception, and female circumcision. See **Table 2** for specific items used to measure these KAN outcomes. The overall KAN outcome was calculated based on “correctly” answering pro-health responses and is indicated by the asterisk in **Table 2**.

**Table 2:** Contingency table comparing endline C’est La Vie viewer (V) and non-viewer (NV) responses to knowledge, attitudes, and norms questions for sexual assault, emergency contraception, and female circumcision*

	Responses	Non-viewer (%)		Viewer (%)	*P*
**Sexual assault (n = 675)**		(n = 470)	(n = 205)		
If someone you know was sexually assaulted, would you encourage them to:					
Talk to someone they trust	Yes†	91.9	95.0		0.097
See a health professional	Yes†	98.6	100.0		0.018
Report to the police	Yes†	93.6	97.9		0.005
In a romantic relationship, one of the partners can be expected to pressure the other for sex.	I agree	25.7	28.7		0.026
	I do not agree†	66.0	67.7		
	I don’t know	8.3	3.6		
**Emergency contraception (n = 551)**		(n = 400)	(n = 151)		
It’s too complicated to use a condom every time you have sex.	I agree	13.5	12.5		0.443
	I do not agree†	60.8	57.7		
	I don’t know	24.3	23.8		
	I have not heard	1.4	6.0		
The use of contraceptive methods is not morally accepted.	I agree	19.2	26.1		0.571
	I do not agree†	61.6	53.4		
	I don’t know	16.4	16.7		
	I have not heard	2.7	3.8		
Using contraceptive methods does not prevent you from getting pregnant.	I agree	39.7	36.3		0.800
	I do not agree†	43.8	44.3		
	I don’t know	13.7	14.1		
	I have not heard	2.7	5.3		
It is mainly up to women to make decisions about birth spacing.	I agree	35.6	47.5		0.202
	I do not agree†	58.9	47.9		
	I don’t know	5.5	3.5		
	I have not heard	0.0	1.2		
Have you ever heard of emergency contraception, also known as the morning after pill? (Aware)	No	10.0	3.6		0.003
	Yes†	90.0	96.4		
When to take emergency contraception to prevent pregnancy? (When)	Anytime	9.4	3.6		<0.001
	During sex	6.9	5.6		
	I don’t know	27.5	14.1		
	Up to 3 d after sex†	53.8	75.5		
	Up to 7 d after sex	2.5	1.3		
When taken correctly, emergency contraception is very effective in preventing pregnancy? (Effective)	False	1.9	4.9		<0.001
	I don’t know	32.5	17.9		
	True†	65.6	77.3		
In your opinion, taking emergency contraception is a socially accepted way to prevent pregnancy? (Accepted)	I agree†	50.6	47.1		0.174
	I do not agree	22.5	30.2		
	I don’t know	26.9	22.8		
I will help a friend get the morning after pill if he or she needs it. (Help)	I agree†	65.6	72.1		0.260
	I do not agree	16.9	15.1		
	I don’t know	17.5	12.8		
**Female circumcision (n = 414)**		(n = 284)	(n = 130)		0.001
In your opinion, should the practice of excision be continued?	I agree	6.7	2.2		
	I do not agree†	88.1	97.1		
	I have not heard	5.2	0.7		
Families should have the right to decide whether to practice FGC if they wish.	I agree	15.7	10.8		0.003
	I do not agree†	75.4	86.7		
	I don’t know	9.0	2.5		
FGC can lead to death.	False	3.0	2.2		<0.001
	I don’t know	16.3	3.9		
	True†	80.7	93.9		

*Column percentages shown. Sample size varied for responses to each section due to number of follow-ups where items were asked: 1) sexual assault (n = 675), emergency contraception (n = 551), and female circumcision (n = 414).

†Indicates “correct” or pro-health response when calculating the overall KAN outcome in Models 1 and 2.

*Key exposure*: Viewing any CLV storyline was the key exposure in our study. To gain a richer understanding of the impact of exposure on KAN outcomes, a secondary exposure was viewing specific storylines (sexual assault, emergency contraception, or female circumcision). In follow-up questionnaires, we included a brief graphical summary of key characters and events for the three separate storylines. If participants remembered seeing the storyline, they were asked questions related to the storyline (see Appendix S1 in the **Online Supplementary Document**).

*Narrative engagement*: If participants had seen a storyline, we assessed their level of engagement. Narrative engagement questions are described in Appendix S2 in the **Online Supplementary Document**. They were modified for each storyline and developed based on existing literature [25]. Engagement levels of low, medium, and high were determined by summing eight items and categorizing the continuous response by standard discretization of varying responses to engagement questions, similar to prior narrative engagement scale development and use [26]. Specifically, responses of “Agree” were coded as “1”, whereas “Do not agree” and “I don’t know” were both coded as “0”. There was one question where subjects were asked how similar the characters were to them, to which they could answer “They are a lot like me” (coded as “2”), “A bit like me” (coded as “1”), or “Not at all like me” (coded as “0”). The eight items were summed and resulted in a 0-9 engagement score that was then binned as: 0-4 = low; 5-7 = medium; and 8-9 = high.

### Data Analysis

We conducted univariate analyses to describe our sample characteristics, and analyses stratified by viewers and non-viewers (**Table 1**). We used bivariate statistics to compare follow-up responses to specific KAN items between viewers and non-viewers (**Table 2**), assessing differences using χ^2^ tests (alpha = 0.05).

To identify the effect of viewing CLV (key exposure) on knowledge, attitudes, and norms (key outcome), we used an item response theory (IRT) approach [27] to develop three models. All covariates described in **Table 1** are controlled for in each of the models presented in **Table 3**. Additionally, all models control for baseline differences in KAN items. For viewers, we also included how many CLV Season 2 episodes the respondent has seen and whether they “liked” or “did not like” the show (variables collected at baseline and not present in **Table 1**). Appendix S3 in the **Online Supplementary Document** describes the development of all study models. Model 1 predicts the effect of seeing vs not seeing any storyline on the odds of providing a pro-health response to a KAN question. Model 2 predicts the effect of self-reported level of viewer engagement (not seen, low, medium, high) on the odds of providing a pro-health response to a KAN question. Model 3 predicts the effect of self-reported level of viewer engagement and takes into account the effect of specific outcome questions on the odds of providing a pro-health response to a KAN question.

**Table 3:** Median (2.5%-97.5%) odds ratio estimates from Models 1, 2, and 3 predicting overall pro-health KAN outcome*

Overall engagement					Specific storyline engagement				
Model 1						**Model 2**		**Model 3†**	
Median		(2.5%-97.5%)				Median	(2.5%-97.5%)	Median	(2.5%-97.5%)
1.26 (1.09-1.45)					**Sexual assault**				
					Low	0.45	(0.23-0.86)	0.43	(0.20-0.87)
					Medium	0.85	(0.67-1.08)	0.84	(0.65-1.10)
					High	1.05	(0.85-1.30)	1.07	(0.85-1.35)
					**Female circumcision**				
					Low	0.79	(0.42-1.47)	0.92	(0.42-2.48)
					Medium	1.19	(0.91-1.55)	1.23	(0.92-1.65)
					High	1.25	(0.97-1.62)	1.22	(0.88-1.69)
					**Emergency contraception**				
					Low	0.96	(0.67-1.37)	0.95	(0.64-1.46)
					Medium	1.29	(1.08-1.56)	1.36	(1.04-1.86)
					High	1.42	(1.18-1.72)	1.38	(1.10-1.74)

*Model 1 fit for overall engagement (seeing vs not seeing storylines), while models 2 and 3 fit across varying levels of engagement with each of the C'est La Vie storylines. Reference category – did not see storylines.

†Model 3 controls for question specific effects.

Exclusively among those who indicated seeing the CLV series, we used logistic regression to predict the odds of a study participant talking to someone else about the CLV series, based on their self-reported level of engagement with the series, and controlling for covariates presented in **Table 1** and **Table 2**.

We fit the models in a Bayesian paradigm and calculated the 90% and 95% posterior credible intervals (CrI) of model parameters, parameter contrasts, probability estimates for specific questions, and levels of subject engagement with the various CLV storylines. Improper priors proportional to 1 were placed on the regression coefficients in the analysis for greater comparability with their frequentist counterparts. Priors placed on the subject and question-specific variance terms were the student *t* test with 3 degrees of freedom and a standard deviation of 2.5 [28]. These priors were used in all models. Additionally, to compare the models’ varying levels of fit, we calculated the difference in expected log predictive density values between Models 1-3 (see Appendix S4 in the **Online Supplementary Document**). All models were fit in R (v.4.0.2). Appendix S3 in the **Online Supplementary Document** provides a more detailed description of model specifications and development.

### Ethics

All study procedures and protocols were approved by Drexel University’s institutional ethical review board (Protocol ID: 00015164).

## RESULTS

### Sample Characteristics

A total of 1674 respondents participated in the study. One in four participants (23%; n = 388) had seen at least one of the three storylines from CLV Season 2 (ie, was designated as a CLV viewer). On average, participants were 24 years old, and viewers were slightly younger (23 years old) than non-viewers (24 years old). Half of the participants were Senegalese, and a higher proportion of viewers (60%) than non-viewers (47%) were Senegalese. Seven in ten participants were Muslim (68%). Most participants were female (60%), and a higher proportion of viewers were female (65%) compared to non-viewers (58%). Nearly nine in ten participants resided primarily in urban areas (88%). Of the 1674 participants who completed the baseline questionnaire, nearly six in ten were lost to follow-up (n = 999; 59.6%).

**Table 2** presents the bivariate analysis of KAN items by CLV viewership. Differences were identified between viewers and non-viewers in KAN items for sexual assault, emergency contraception, and female circumcision. Viewers were more likely than non-viewers to agree that victims of sexual assault should report it to the police (*P* = 0.005), to have heard about emergency contraception (*P* = 0.003), and to know when to correctly use emergency contraception (*P* < 0.001). Viewers were also significantly more likely to believe that the practice of female circumcision should end (*P* = 0.001) and that it can lead to death (*P* < 0.001).

*RQ1: Overall engagement effects on overall KAN outcome:* In Model 1 (see **Table 3**) we estimated the odds ratio of improved KAN response as a function of viewing any CLV storyline vs not viewing CLV to be 1.26 (95% CrI = 1.09-1.45). This implies that, on average, viewers who saw any CLV storyline had 26% higher odds of correctly answering a KAN question (overall, not specific questions) than respondents who did not see a storyline, controlling for covariates described in **Table 1**.

*RQ2: Specific storyline engagement effects on overall outcome questions:* Model 2 in **Table 3** shows that higher levels of self-reported engagement corresponded to greater odds of answering a KAN question correctly. However, this association between different levels of engagement and the odds of correctly answering a question varied substantially between storylines. Those who reported higher levels of engagement with the emergency contraception storyline experienced the greatest levels of improvement in their ability to correctly answer KAN questions, with an odds ratio of 1.42 (95% CrI = 1.18-1.72) amongst those who reported “High” engagement compared to respondents who had not seen the storyline. In contrast, those who reported varying levels of engagement with the sexual assault storyline only ever reported a credibly decreased likelihood of correctly answering a KAN question, OR = 0.45 (95% CrI = 0.23-0.86), amongst those who reported “Low” engagement and a non-credible association otherwise.

**Figure 1:**
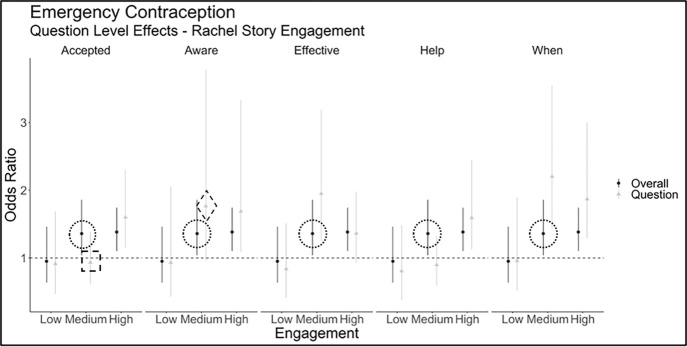
Point and lines represent median and 95% credible interval estimates of the odds ratio of estimating the overall outcome question (dark point estimate, same across all specific questions in this figure) and the specific question (light triangle estimate) across varying levels of engagement with the emergency contraception story. Full text for the question (ie, accepted, aware, etc.) can be found in **Table 2****.**

*RQ3: Specific storyline engagement effects on specific outcome questions:* For Model 3 in **Table 3**, we present the overall KAN odds ratio estimates controlling for question specific effects. While there were few differences in odds ratio estimates between the overall KAN outcome and specific question outcomes (eg, “In your opinion, taking emergency contraception is a socially accepted way to prevent pregnancy”) there was a subset of questions for which the question-specific effects were different from the overall effects. In **Figure 1**, we highlight a subset of questions specific to emergency contraception as these showed the greatest difference from the overall KAN outcome. The questions are presented in **Table 2** and indicated with the marker (eg, accepted, ware, etc.). Both overall KAN outcome (black point estimates in **Figure 1**) and question-specific effects (grey triangle point estimates in **Figure 1**) highlight the potential amplification or attenuation of KAN associations with CLV engagement.

Specifically, the overall odds ratio of answering a KAN outcome correctly for medium engagement with the emergency contraception storyline, compared to answering it without seeing the storyline at all, was estimated to be 1.36 (95% CrI = 1.04-1.86), shown by the dotted circles in **Figure 1** (values presented in **Table 3**). When examining question-specific effects, this relationship changes. For example, when predicting the “Accepted” question (“In your opinion, taking emergency contraception is a socially accepted way to prevent pregnancy”) for medium engagement, the relationship is attenuated to 0.796 (95% CrI = 0.55-1.15), shown by the dashed square in **Figure 1**. In contrast, when predicting the “Aware” question (“Have you ever heard of the emergency contraception, also known as the morning after pill?”), the same level of engagement resulted in an amplified or increased odds of correct response with an estimate of 2.21 (95% CrI = 1.23-4.55), shown by the dashed diamond in **Figure 1**. While most of the question-specific outcomes followed the same pattern as the overall KAN outcome, a few demonstrated an increased (or in some cases a decreased) effect of viewing the CLV story.

**Table 4** presents the estimates of the contrasts in these probability estimates, to determine whether different levels of engagement have credibly different effects (ie, comparing low to medium engagement, or medium to high engagement). The high posterior probability values provide evidence that amongst those individuals who watched a given storyline, those reporting more engagement were more likely to answer a typical KAN question correctly. For example, in Model 2 under the emergency contraception story, we see a credible difference of 0.83 between medium and high engagement; that is, there is an 83% probability that high engagement has a credibly higher association with a typical KAN outcome compared to medium engagement. Model 3 has the best fit when comparing the difference in expected log pointwise predictive density results (see Appendix S4 in the **Online Supplementary Document**). This suggests that accounting for differences in storyline across different question responses explains the observed variability in responses better than the alternative.

**Table 4:** Posterior probability of the denoted parameter contrast for each of the two models fit

		Model	
		**Model 2**	**Model 3**
**Sexual assault storyline engagement levels**			
Medium>Low		0.97	0.96
High>Low		0.99	0.99
High>Medium		0.94	0.94
**Female circumcision storyline engagement levels**			
Medium>Low		0.90	0.72
High>Low		0.92	0.72
High>Medium		0.64	0.49
**Emergency contraception storyline engagement levels**			
Medium>Low		0.95	0.93
High>Low		0.98	0.95
High>Medium		0.83	0.54

*RQ4: Storyline engagement effects on talking about the CLV series with someone:* We identified a strong association between respondents who reported medium or high engagement and their self-reported sharing of the CLV series with others. Individuals responding with medium engagement had 10.2 (95% CrI = 2.9-42.0) greater odds to say they talked about the CLV series than an individual with low engagement, while an individual reporting high engagement had 23.2 (95% CrI = 6.5-93.4) greater odds of talking to someone about the CLV series than an individual with low engagement. RQ4 results are not shown in a table (see Appendix S3 in the **Online Supplementary Document** for Model 4 specifications)

## DISCUSSION

Our findings demonstrate that online health EE, particularly pro-health content that engages audiences with captivating stories, can lead to positive changes in KAN among netizens in francophone West Africa. Watching the CLV series online had an impact on overall KAN outcomes when compared to not watching CLV; viewers of the series had 26% greater odds of answering pro-health responses about sexual assault, emergency contraception, and female circumcision. Further, the level of engagement with specific storylines was associated with a differential impact on overall outcome questions, most notably with the storyline about emergency contraception.

Online content, including web series and social media posts, can and should be developed for communities around the globe, particularly as access to digital technologies continues to grow. In addition, our findings demonstrate the effectiveness and potential impact of making content that is developed for more traditional media available through online platforms. We evaluated C’est La Vie content that was originally developed for broadcast media and then made available for online consumption. Not only does providing content online expand potential audience reach, but it also provides opportunities for audiences to engage with content in ways that would only be possible online (ie, through commenting or sharing of content). Traditionally, social media and other online content have been developed in shorter format to be consumed very quickly; however, our findings show that longer-format content, such as mini-series that are compelling and tap into storylines and emotions, can have an impact on the audience’s health-related behaviours.

This study further provides evidence that health content delivered through entertaining storylines can lead to positive pro-health changes among audiences in West Africa. Specifically, when audiences identify or empathize with content (ie, report high levels of engagement) there are greater changes in pro-health KAN. Furthermore, our findings demonstrated a differential impact on KAN outcomes based on different storylines. In our study, the emergency contraception storyline was associated with the greatest odds of answering pro-health KAN outcomes, whereas the sexual assault storyline was associated with more modest or even lower odds of answering pro-health KAN outcomes. While the emergency contraception storyline featured more prominent and younger characters compared to the sexual assault storyline that featured more minor characters, we are unable to say exactly what part of the story was more engaging, be it the characters, the writing, the production, or the topic. This warrants further investigation in future studies. While our findings contribute to a robust body of existing literature and research related to EE [5], and its value in delivering health content to wide audiences, we also add to a growing body of literature examining this effect in online environments [8], specifically, in West Africa.

Our approach to evaluating the impact of online health content on audiences in West Africa provides a novel set of tools and a roadmap for future work in this field. We demonstrate the feasibility and describe the approach to recruiting online participants in francophone West Africa to evaluate a health program, utilizing two social media platforms that are widely available in much of the world. Additional social media platforms and other online venues should be explored as access and coverage continue to expand around the globe. While novel online platforms emerge regularly, our approach and methods can and should be adopted and adapted to meet the needs and nuances of new media environments. Furthermore, as health content becomes more interactive and, in many cases, created by audiences themselves, evaluation methods, too, must adapt and incorporate audience-created content and evaluation metrics on its impact on health outcomes.

Our study has several limitations worth noting. First, results rely on self-reported KAN data and do not represent changes in behaviours. While examining behaviour change would add to our findings, such as the use of emergency contraception, our survey methodology provides insights into KAN that are important pathways to behaviour change, often necessary but insufficient on their own. Second, as health content and data collection occurred online, access to the internet (either by mobile device, computer, or other technology) was required for participation. Thus, the priority population in this study may represent a more educated group compared to the general population. However, access to the internet and other digital technologies will continue to grow in traditionally under-resourced areas around the globe and describing our evaluation approach will be useful in the next phase of this research. Third, determination of the key exposure – having seen CLV online content or not – could be subject to recall bias. To minimize the potential for recall bias, we presented all study participants, at each follow-up, with a short story synopsis and visual of characters to ask if they had ever seen the content. This was also used to identify and account for potential crossover between viewers and non-viewers. If a participant “crossed over” it would materialize in the data as them having viewed the storyline at one of the follow-ups. Our models estimate the effect of any participant who reported viewing a storyline on their KAN. Their information would be included in the effect estimate at that point and not prior. Finally, while we provided incentives and attempted to contact participants multiple times through various means (eg, email, WhatsApp, Facebook) throughout the study period, loss to follow-up was substantial. This may introduce bias based on attrition, for reasons we were unable to measure including difficulties with internet access, lack of interest, or some other explanation. We hope that, by noting this limitation, future work in this field, particularly in the digital space, can anticipate and innovate with methodological advancements to strengthen participant follow-up.

## CONCLUSIONS

To the best of our knowledge, our study is one of very few that assesses storyline impact of a health-focused drama among online audiences in West Africa. As internet access continues to grow across the globe and health education materials are created and adapted for new media environments, our study provides a novel approach to examining the impact of online health education strategies. As the line between online content creators and consumers becomes more blurred and the interactive nature of social media continues to drive engagement, our hope is that this study provides evidence for continued work in this field around the globe.

## Additional material


Online Supplementary Document
