# Supplementary material for: Measuring impact of storyline engagement on health knowledge, attitudes, and norms: A digital evaluation of an online health-focused serial drama in West Africa
Source: J Glob Health. 2022 May 14;12:04039. doi: 10.7189/jogh.12.04039 (PMC9107188; doi:10.7189/jogh.12.04039)
Supplement: Online Supplementary Document [file jogh-12-04039-s001.pdf]

Appendix S1. Graphical summaries used to prompt respondents for the three storylines evaluated: Sexual assault, emergency contraception, and female circumcision. After participants reviewed these graphical summaries, they were asked if they had seen this storyline. If they answered yes to seeing any of the storylines, they were treated as viewers; if they answered no to all storylines, they were treated as non-viewers.

Sexual assault

In this storyline from "C'est La Vie!":

- Aïsha is sexually assaulted by a group of unknown men.
- Aïsha tell Rachel about the assault.
- Rachel accompanies Aïsha to the health center.
- At the health center, Aïsha is treated by Magar and Dr Moulaye.

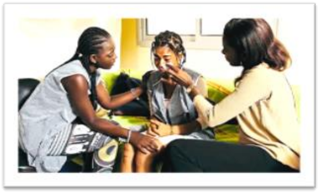

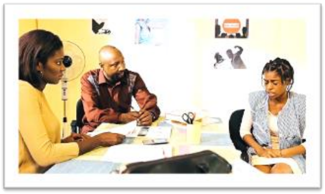

Emergency contraception

In this storyline from "C'est La Vie!":

- Rachel discusses sexual intercourse and consent with Magar.
- Rachel and Julien discuss consent before having sex.
- Rachel and Julien forgot to use a condom during sex.
- Rachel visits multiple pharmacies in search of the emergency contraception pill.

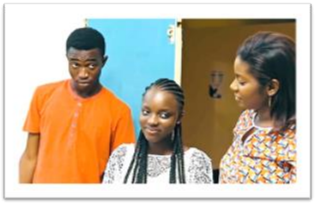

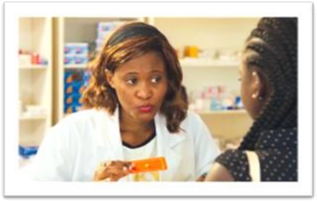

Female circumcision

In this storyline from "C'est La Vie!":

- There is a trial to determine who is responsible for the death of Magar's daughter, Caro, who died in Season 1 from complications related to female circumcision.
- Rokoba, Touli, and la Féticheuse [FGM practitioner] are defendants.
- The first trial takes place in the village of Jolal.
- The second trial takes place in the city of Ratanga.

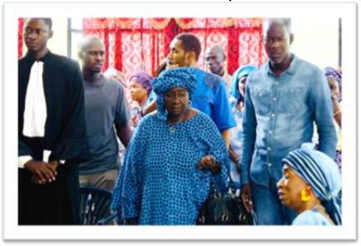

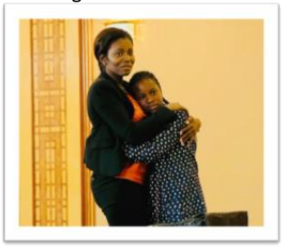

Appendix S2. Narrative engagement questions and response options. Asterisk (*) indicates item used in engagement scale to create three categories: low, medium, high.

Question	Responses	Sexual assault, n=205 (%)	Emergency contraception, n=151 (%)	Female circumcision, n=130 (%)

*I found myself thinking of ways the story could have turned out differently.	I agree	61.0	70.1	70.4
	I do not agree	23.5	21.8	25.5
	I don't know	15.5	8.1	4.1
*I was so caught up in the story that I forgot what was going on in my own life.	I agree	64.9	51.3	61.5
	I do not agree	28.1	41.6	33.3
	I don't know	7.0	7.1	5.1
*While the program was on I found myself thinking about other things.	I agree	22.6	27.1	28.5
	I do not agree	70.9	65.9	67.4
	I don't know	6.5	7.1	4.2
*I could easily follow the action and events.	I agree	82.5	85.0	85.4
	I do not agree	10.0	9.4	10.4
	I don't know	7.5	5.6	4.2
*The story affected me emotionally.	I agree	95.0	68.1	92.2
	I do not agree	3.1	26.3	4.2
	I don't know	2.0	5.6	3.7
*During the program, when a main character felt happy, I felt happy, and when they suffered in some way, I felt sad.	I agree	89.6	83.5	90.1
	I do not agree	7.0	10.9	6.8
	I don't know	3.4	5.6	3.1
*My perception of [story topic] matches the story in the series.	I agree	81.1	75.8	84.1
	I do not agree	10.5	15.0	9.2
	I don't know	8.5	9.1	6.7
*The characters involved in this story are:	A bit like me or people I know	49.3	53.2	34.6
	A lot like me or people I know	27.6	26.5	27.7
	Not at all like me or people I know	23.1	20.3	37.8
After watching this storyline, did you talk to anyone about it?	I intend to	24.1	14.6	11.5
	No	27.9	33.2	30.0
	Yes	48.0	52.2	58.5

Appendix S3: Item Response Theory Approach

We model the CLV survey respondent data using an item response theory (IRT) approach. Specifically, for the **p**th respondent (**p=1,...,1674**) at the **j**th (j=0,1,2,3) visit we map the **i**th (**i**=1,...,21) survey item response to a {0,1} scale, creating the outcome variable Y_{ipj}. Y_{ipj}=1 indicates the respondent has ``correctly'' answered some question about KAN and Y_{ipj}=0 indicates they either did not know, or incorrectly answered the question. Our modeling objective will be to analyze the probability $\pi_{ipj} = E[Y_{ipj}|-]$ conditional on covariates and latent terms we now introduce.

We include all subject level demographic information (see Table 1 variables) including a quadratic expansion of age at baseline, dummy variables for self-reported sex and education. We also included how many episodes of CLV Season 2 the respondent has seen and whether they like or do not like the show (variables not present in Table 1). This includes. We also include the time from baseline at which the subject has filled the survey in order to allow for comparisons with respondents across time and decompose the time-varying ``how much do you like CLV'' response into between and within subject components to estimate these effects separately.

Defining these variables in notation, let α be a global intercept and $\mathbf{Z_{pj}}$ the vector containing all aforementioned subject-level covariates at measurement **j** and their corresponding regression coefficients $\boldsymbol{\delta}$. Let $X_{ij}^{(s+)}$ be the {0,1} variable indicating subject **i** viewed any CLV storyline at follow-up **j** and $\beta^{(s+)}$, it's corresponding effect. Similarly, for Model (2), let $X_{ij}^{(s,k)}$ be the {0,1} variable indicating that subject **i** reports having engagement level **k** with story line **s** at measurement **j** and $\beta^{(s,k)}$ its corresponding effect. Denoting b_i and b_p as item and subject level latent intercepts, respectively, we complete our model specifications by linking covariates via the logistic function:

$$\text{logit}(\pi_{ipj}) = \alpha + \boldsymbol{Z}_{pj}^T\boldsymbol{\delta} + \beta^{s+}X_{pj}^{s+} + b_i + b_p, \qquad \text{(Model 1)}$$

$$\text{logit}(\pi_{ipj}) = \alpha + \boldsymbol{Z}_{pj}^T\boldsymbol{\delta} + \sum_{k=2}^{4}\sum_{s=1}^{3}\beta^{(s,k)}X_{pj}^{(s,k)} + b_i + b_p. \qquad \text{(Model 2)}$$

Both b_i and b_p are modeled as parameters drawn from independent normal distributions with unknown variance: e.g. $b_i \sim N(0,s^2_{\text{ item}})$. In the IRT literature, b_i is referred to as the latent question difficulty and b_p as the latent subject knowledge of whatever construct the items are designed to measure. In our context the latter would be an overall understanding of normative KAN after accounting for all subject covariates. In accordance with our goal of trying to understand the effect of public health media on KAN, the estimation of $\beta^{(s+)}$ in Model 1 and $\beta^{(s,k)}$, (**s** = 1,2,3,**k** = 2,3,4) in Model 2 represent our primary goal of inference.

In Model 3, $\mathbf{b_{sk}}$ represents the vector of question level effects specific to the **s**th storyline and the **k**th level of engagement, and b_{io} is the intercept term representing the item difficulty for individuals who have not seen any CLV storyline.

$$\begin{aligned}\text{logit}(\pi_{ipj}) = \alpha + \boldsymbol{Z}_{pj}^T\boldsymbol{\delta} + \sum_{k=2}^{4}\sum_{s=1}^{3}\beta^{(s,k)}X_{pj}^{(s,k)} + b_p + \qquad \text{(Model 3)}\\ b_{i0} + \sum_{s=1}^{3}\sum_{k=2}^{4} b_{isk}X_{pj}^{(s,k)},\\ \boldsymbol{b}_{sk} \overset{iid}{\sim} MVN_{21}(\mathbf{0}, \sigma_{sk}\boldsymbol{I}).\end{aligned}$$

In Model 4, we now estimate the probability of survey respondent, **p (p=1,…,388)**, at measurement **j (j = 2,3),** who has seen storyline **s (s=1,2,3),** responding that they would tell someone about the CLV series. We denote this probability as π'_{pjs} and model this entity as a function of the same subject level covariates and effects discussed previously, $\mathbf{Z}_{pj}$ and $\boldsymbol{\delta}$, respectively. Similar to before, we include a {0,1} indicator measurement for the subject having the **k**th engagement level with the storyline **s**, denoted $\mathbf{X_{pjs}^{(k)}}$.

The exponential of $\beta^{(k)}$, the odds ratio, represents our inferential objective. We complete our model specification by including a subject-specific intercept b_p, to adjust for within subject correlation across the follow-ups surveys:

$$\text{logit}(\pi'_{pjs}) = \alpha' + \boldsymbol{Z}_{pj}^{T}\boldsymbol{\delta} + \sum_{k=1}^{2} \beta^{(k)} X_{pjs}^{(k)} + b_p \qquad \text{(Model 4)}$$

$$b_p \sim N(0, \sigma_{person}^2).$$

It should be noted that we explored models that would allow for different odds of sharing as a function of storyline and engagement interactions but these effects were found to non-credibly different from zero. Thus, we present and discuss Model 4 for simplicity.

As a final note, while all estimates of differences made here are explicitly between those who have seen CLV at some point during follow-up and those who had not seen CLV at baseline, this comparison could be adjusted by including the desired time point for comparison and multiplying by the time-slope. As this time slope was estimated to be effectively – and credibly – zero in all models, we resort to the simplest comparison, between individuals who had seen a specific storyline during follow up and all those who had not at baseline.

Appendix S4. Model comparison using expected log pointwise predictive density

Model	Δ elpd*	Standard error Δelpd
3	0	0
2	-18.87	7.92
1	-20.85	9.30

**Elpd refers to expected log predictive density a measure of model performance. Δ elpd refers to the difference in expected log predictive density between the best performing model (Δ elpd = 0) and subsequently performing models.*
